# Ketone body and FGF21 coordinately regulate fasting-induced oxidative stress response in the heart

**DOI:** 10.1038/s41598-022-10993-4

**Published:** 2022-05-05

**Authors:** Ryo Kawakami, Hiroaki Sunaga, Tatsuya Iso, Ryosuke Kaneko, Norimichi Koitabashi, Masaru Obokata, Tomonari Harada, Hiroki Matsui, Tomoyuki Yokoyama, Masahiko Kurabayashi

**Affiliations:** 1 Department of Cardiovascular Medicine, Gunma University Graduate School of Medicine, 3-39-15 Showa-machi, Maebashi, Gunma 371-8511 Japan; 2 Center for Liberal Arts and Sciences, Ashikaga University, 268-1 Omae-machi, Ashikaga, Tochigi 326-8558 Japan; 3 Bioresource Center, Gunma University, Graduate School of Medicine, Maebashi, Gunma Japan; 4 Osaka University, Graduate School of Frontier Biosciences, 1-3 Yamadaoka, Suita, Osaka Japan; 5 Department of Laboratory Sciences, Gunma University Graduate School of Health Sciences, Maebashi, Gunma Japan

**Keywords:** Cardiology, Cardiovascular biology, Molecular biology, Transcription

## Abstract

Ketone body β-hydroxybutyrate (βOHB) and fibroblast growth factor-21 (FGF21) have been proposed to mediate systemic metabolic response to fasting. However, it remains elusive about the signaling elicited by ketone and FGF21 in the heart. Stimulation of neonatal rat cardiomyocytes with βOHB and FGF21 induced peroxisome proliferator-activated receptor α (PPARα) and PGC1α expression along with the phosphorylation of LKB1 and AMPK. βOHB and FGF21 induced transcription of peroxisome proliferator-activated receptor response element (PPRE)-containing genes through an activation of PPARα. Additionally, βOHB and FGF21 induced the expression of Nrf2, a master regulator for oxidative stress response, and catalase and Ucp2 genes. We evaluated the oxidative stress response gene expression after 24 h fast in global Fgf21-null (Fgf21^−/−^) mice, cardiomyocyte-specific FGF21-null (cmFgf21^−/−^) mice, wild-type (WT), and Fgf21^fl/fl^ littermates. Fgf21^−/−^ mice but not cmFgf21^−/−^ mice had unexpectedly higher serum βOHB levels, and higher expression levels of PPARα and oxidative stress response genes than WT mice or Fgf21^fl/fl^ littermates. Notably, expression levels of oxidative stress response genes were significantly correlated with serum βOHB and PGC1α levels in both WT and Fgf21^−/−^ mice. These findings suggest that fasting-induced βOHB and circulating FGF21 coordinately regulate oxidative stress response gene expression in the heart.

## Introduction

The heart is the most energy-demanding and metabolically omnivorous organ which uses ketone bodies as well as fatty acids and glucose as fuel source^1^. It is a growing appreciation that circulating ketone body, β-hydroxybutyrate (βOHB), is not just used during fasting and exercise, but also has important cellular signaling roles to regulate gene expression^2,3^. Histone acetylation is important for the global transcription and specific changes in gene expression^4^. Because βOHB is catabolized to acetyl-CoA in target tissues, metabolism of βOHB into acetyl-CoA should raise intracellular acetyl-CoA levels, and increase the acetylation of histones and non-histone proteins which are involved in several cellular processes controlling anabolic and catabolic reactions during fasting response^5^. Indeed, it was discovered that βOHB represses oxidative stress in mouse kidney by inhibiting endogenous class I histone deacetylase (HDACs) activity^6^. In addition, lysine β-hydroxybutyrylation was found to be distinct βOHB-derived histone modification in cultured cells and in the liver from mice subjected to prolonged fasting^7^. In line with this notion, βOHB may have far-reaching effect on overall metabolic health, given that nutrient sensitive pathways activated in calorie restriction, intermittent fasting and ketogenic diets have important roles in metabolic homeostasis and longevity^3^. However, the precise signaling pathways by which βOHB regulates the cardiac gene expression remain largely unknown.

Fibroblast growth factor 21 (FGF21) is a metabolic hormone that is induced during fasting and exercise in mice and humans^8^. While the main site of production and release into the circulation is the liver^9^, FGF21 is also produced and released from skeletal muscle and cardiac muscle^10–12^. Besides an induction of glucose uptake in adipocytes^13^ and thermogenic activation^14^, FGF21 increases lipolysis in white adipose tissue^15,16^ and ketogenesis on ketogenic diet^17^. An induction of FGF21 expression after fasting or by consumption of ketogenic diet is in large part under the control of peroxisome proliferator-activated receptor α (PPARα)^15,17^.

PPARα, a member of a family of related nuclear receptors including PPARδ (also known as PPARβ) and PPARγ, is a key regulator of energy metabolism in the liver and in the heart^18^; the PPARα-null mice have lower mitochondrial fatty acid oxidation (FAO) rate in the heart and have impaired adaptive response to fasting or ingestion of a ketogenic diet in the liver^19^. Taken together, the results of previous studies suggest that PPARα plays a major role in the production of βOHB and FGF21 in the liver as an adaptive response to fasting. However, it remains largely unknown about the signaling pathways that might mediate the effects of βOHB and FGF21 on the cardiac metabolism during fasting.

In this study, we demonstrate that βOHB and FGF21 induce PPARα expression and its trans-activating function in cultured cardiac myocytes. Global Fgf21-null (Fgf21^−/−^) mice but not cardiac myocyte-specific Fgf21 knockout (cmFgf21^−/−^) mice unexpectedly exhibited higher serum βOHB levels and higher expression levels of PPARα, and oxidative stress response gene expression after 24 h fasting than control mice. Our findings suggest that circulating βOHB and FGF21 coordinately regulate fasting-induced oxidative stress response in the heart.

## Results

### Stimulation of metabolic gene expression by βOHB and FGF21 in cardiac myocytes

**Figure 1:**
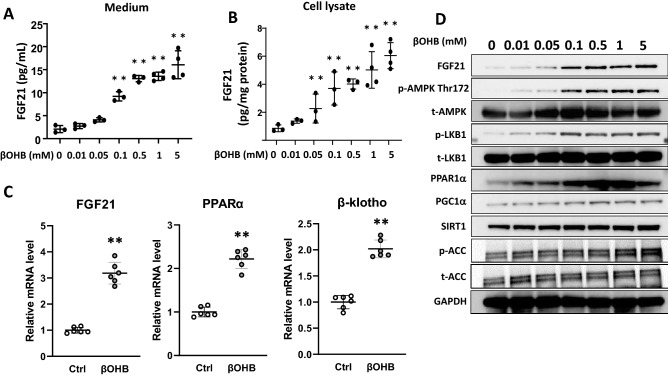
Effects of βOHB on FGF21 expression and signaling in cultured neonatal rat cardiac myocytes. FGF21 concentrations in cultured medium (**A**) and in cell lysates (**B**) were measured by ELISA after stimulation of cultured neonatal rat cardiac myocytes with βOHB (0–5 mM) for 24 h Data are mean ± SD (n = 3–4). **p < 0.01 vs control (0 mM) analyzed by one-way ANOVA followed by Dunnett correction for multiple comparisons. (**C**) mRNA levels for FGF21, PPARα and β-klotho were compared between the vehicle (ctrl) and βOHB (0.5 mM)-treated cardiac myocytes (n = 6). Data are mean ± SD (n = 6). **p < 0.01 vs control analyzed by unpaired Student’s t-test. (**D**) Representative western blots to assess the relative changes of FGF21 and indicated signaling molecules in response to βOHB at indicated concentrations for 24 h. Full-length blots are presented in Supplementary Fig. S1.

To determine whether βOHB regulates the signaling pathways relevant to the cardiac metabolism, we examined mRNA and protein levels of FGF21 and other molecules which are known to play a role in energy metabolism and hypertrophic response using the cultured neonatal rat cardiac myocytes. The results of ELISA showed that βOHB increased the FGF21 concentrations in the culture medium as well as in the cell lysates in a dose-dependent manner (Fig. 1A,B). Quantitative real-time PCR (qPCR) showed that βOHB (0.5 mM) induced FGF21 and PPARα by 3.2-fold (p < 0.01) and 2.2-fold (p < 0.01), respectively. Interestingly, βOHB induced the expression of the β-klotho gene, a co-receptor of FGF21, by 2.0-fold (p < 0.01) (Fig. 1C). Expression levels of the genes coding for the enzymes involved in the oxidation of ketone body, 3-hydroxybutyrate dehydrogenase (Bdh1) and succinyl-CoA:3-ketoacid coenzyme A transferase (Oxct1, also known as SCOT), were not altered by βOHB.

Western blot analysis revealed that βOHB induced the FGF21 and PPARα protein levels in a dose-dependent manner (Fig. 1D). βOHB increased the phosphorylation of Thr^172^ of AMP-activated protein kinase (AMPK), phosphorylation of Ser^428^ of LKB1, a tumor suppressor and a key regulator of AMPK activation^20,21^, peroxisome proliferator-activated receptor-coactivator 1α (PGC1α), and Sirt1. In addition, βOHB increased the phosphorylation of acetyl-CoA carboxylase (ACC) at Ser^79^, an authentic phosphorylation site by AMPK^22^. Full-length representative western blots are shown in Supplementary Fig. S1. These results suggest that βOHB activates signaling pathway which is known to be activated when the cells were exposed to nutrient stress with the ensuring induction of the FGF21 gene in cardiac myocytes.

### Stimulation of FGF21, PPARα and β-klotho gene expression by FGF21

**Figure 2:**
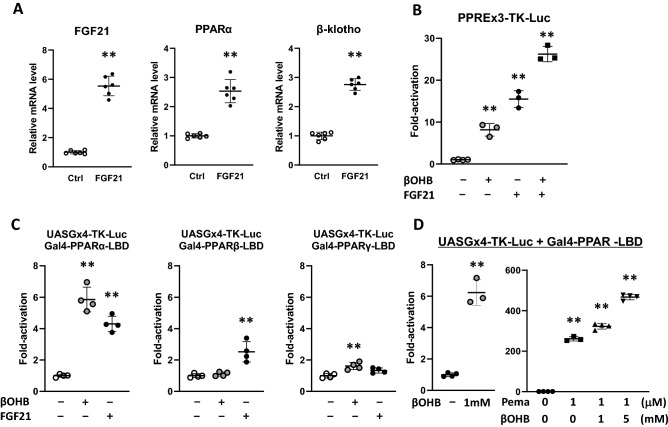
Effects of FGF21 on signaling molecules and transactivating function of PPARα. (**A**) mRNA levels for FGF21, PPARα and β-klotho were compared between the vehicle (ctrl) and FGF21 (10 ng/mL)-treated cardiac myocytes. Data are mean ± SD (n = 6). **p < 0.01 vs control analyzed by unpaired Student’s t-test. (**B**) Cultured neonatal rat ventricular cardiac myocytes were transfected with the reporter constructs contained three copies of PPRE in front of TK promoter. Following transfection, cells were treated with vehicle, 0.5 mM of βOHB, or 10 ng/mL of FGF21 for 24 h before harvest for luciferase activity assay. Luciferase activity was plotted as fold-activation relative to untreated control cells. Data are mean ± SD (n = 3–4). **p < 0.01 vs control analyzed by one-way ANOVA followed by Dunnett correction for multiple comparisons. (**C**) Cultured neonatal rat ventricular cardiac myocytes were transfected with the reporter constructs containing four copies of the UAS_G_ cloned upstream of the TK-Luc reporter (UAS_G_x4-TK-Luc) together with CMX-Gal-PPARα, CMX-Gal-PPARδ-, or CMX-Gal-PPARγ-LBD. Following transfection, cells were treated with vehicle, 0.5 mM of βOHB, or 10 ng/mL of FGF21 for 24 h before harvest for luciferase activity assay. Luciferase activity was plotted as fold-activation relative to untreated control cells. Data are mean ± SD (n = 4). **p < 0.01 vs control analyzed by one-way ANOVA followed by Dunnett correction for multiple comparisons. (**D**) Neonatal rat ventricular cardiac myocytes transfected with UAS_G_x4-TK-Luc and CMX-Gal-PPARα were stimulated by βOHB and/or Pemafibrate. Data are mean ± SD (n = 3–4). **p < 0.01 vs control analyzed by unpaired Student’s t-test (left panel) and one-way ANOVA followed by Dunnett correction for multiple comparisons (right panel).

The finding that FGF21 is released from cardiac myocytes prompted us to test whether FGF21 elicits the signaling in cardiac myocytes. qPCR showed that FGF21 (10 ng/ml) induced FGF21, PPARα, and β-klotho expression by 5.9-fold (p < 0.01), 2.6-fold (p < 0.01), and 2.9-fold (p < 0.01) respectively (Fig. 2A). These results indicate that FGF21 evokes a signal to induce its own expression as well as PPARα and β-klotho gene expression in the cardiac myocytes.

### Stimulation of PPRE-driven transcription by βOHB and FGF21

Previous studies showed that FGF21 expression is regulated by PPAR-responsive element (PPRE) in the liver^15^. To test whether this is also true for cardiac myocytes, we performed transient transfection assays of the reporter plasmid PPREx3-TK-Luc, which contains three copies of authentic PPRE in front of the TK gene^23^. Cardiac myocytes transfected with this plasmid were stimulated with either βOHB, FGF21 or both for 24 h. Result showed that βOHB, FGF21 or both significantly induced PPREx3-TK luciferase activity by 9.0-fold, 15.1-fold, and 26.5-fold, respectively (Fig. 2B). These results suggest that each of βOHB and FGF21 stimulates PPRE-driven transcription, and additive induction of reporter genes by simultaneous stimulation suggests that βOHB and FGF21 induce PPRE via a common or overlapping signaling pathway in the cardiac myocytes.

### Stimulation of PPARα activity by βOHB and FGF21

Next, we asked whether βOHB and FGF21 increase trans-activating function of PPARα. We transfected cardiac myocytes with a Gal4 reporter construct, UAS_G_x4-TK-Luc that contains four copies of Gal4 upstream activating sequence (UAS_G_) in front of thymidine kinase (TK) gene promoter, and a vector expressing Gal4-mPPARα-LBD fusion protein in which the DNA binding domain (DBD) of Gal4 is linked to the ligand binding domain (LBD) of the mouse PPARα^23^. These chimeric receptors allow us to assay the transactivating function of PPARα independently of the endogenous receptors. The transfected cells were stimulated with βOHB (Fig. 2C). Results showed that βOHB increased the luciferase activity driven by Gal4-PPARα-LBD by 5.8-fold. In contrast, the luciferase activities driven by Gal4-PPARδ-LBD and Gal4-PPARγ-LBD, in which Gal-DBD is linked to PPARδ-LBD and PPARγ-LBD, respectively, were activated to a much lesser extent (Fig. 2C). These results suggest that βOHB preferentially activates PPARα through an LBD-dependent mechanism. Similarly, FGF21 significantly increased PPARα-LBD-driven luciferase activity. FGF21 seemed to weakly increase PPARδ-LBD-driven activity. These results suggest that βOHB and FGF21 are preferential activators of the α isoform of PPAR.

We next examined the possible interaction between βOHB and authentic PPARα ligand to activate PPARα-dependent transcription. We used pemafibrate as a bona fide PPARα ligand. Pemafibrate is known as a selective PPARα modulator (SPPARMα), and has widely been used for the patients with hypertriglyceridemia^24^. We found that 1 mM βOHB and 1 µM of pemafibrate increased the luciferase activity driven by Gal4-PPARα-LBD by 6.2-fold and 260-fold, respectively. Interestingly, stimulation of cardiac myocytes with both βOHB and pemafibrate resulted in robust increase in luciferase activity. Combination of 1 µM of pemafibrate and 1 mM of βOHB induced 300-fold activation, and combination of 1 µM of pemafibrate and 5 mM of βOHB induced 420-fold activation. Activation of luciferase activity to a greater extent than mere sum of the individual activation by βOHB and pemafibrate suggests that βOHB induces activation of PPARα function through the distinct mechanisms by which pemafibrate activates PPARα function (Fig. 2D).

### Upregulation of the oxidative stress response genes and downregulation of hypertrophic marker genes by βOHB and FGF21

**Figure 3:**
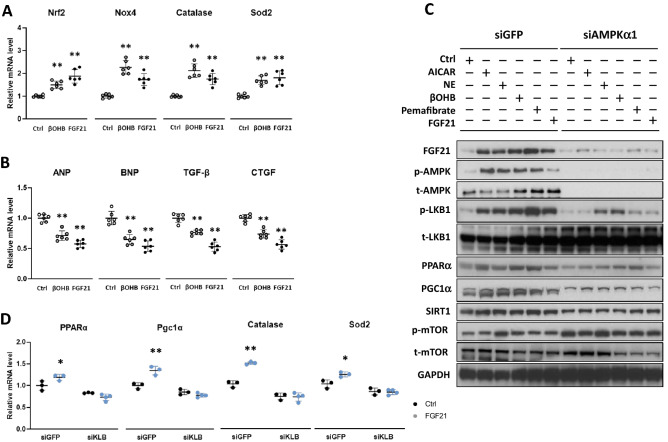
Effects of βOHB and FGF21 on the metabolism-related genes and proteins in the cultured cardiac myocytes. qPCR was performed to measure the expression levels for the oxidative response genes (**A**) or hypertrophic marker genes (**B**) after stimulation of the cells with βOHB (0.5 mM) and FGF21 (10 ng/mL). mRNA levels in stimulated cells were plotted relative to untreated control cells. Data are mean ± SD (n = 6). **p < 0.01 vs control analyzed by one-way ANOVA followed by Dunnett correction for multiple comparisons. (**C**) Representative western blots of indicated proteins. Neonatal rat ventricular cardiomyocytes were transduced with either siRNA for GFP or AMPKα1 for 24 h, and then cells were treated with either 1 mM of 5-aminoimidazole-4-carboxamide ribonucleotide (AICAR), a cell-permeable adenosine analogue, AMPK activator, norepinephrine (NE 10 µM), βOHB (0.5 mM), pemafibrate (10 µM), or FGF21 (10 ng/mL) for 24 h before harvest for western blot analysis. Full-length blots are presented in Supplementary Fig. S2. (**D**) Neonatal rat ventricular cardiac myocytes were transduced with either siRNAs for GFP or β-klotho (KLB) for 24 h, and then treated with 10 ng/mL of FGF21 for 24 h before harvest for qPCR analysis. Data are mean ± SD (n = 3–4). *p < 0.05 and **p < 0.01 vs corresponding control analyzed by one-way ANOVA followed by Tukey correction for multiple comparisons.

Antioxidant defense mechanisms are regulated by a complex network of antioxidant genes^25^. We next explored the involvement of βOHB and FGF21 in the regulation of the gene expression relevant to oxidative stress response in cardiac myocytes by qPCR (Fig. 3A). βOHB significantly induced the nuclear factor erythroid-derived 2-like 2 (Nrf2), which activates transcription of a variety of cytoprotective genes by binding to a well-defined antioxidant response element (ARE) sequence^26,27^. βOHB also induced mitochondrial ROS-generating enzyme, nicotinamide adenine dinucleotide phosphate (NADPH) oxidases 4 (Nox4), catalase, and mitochondrial antioxidative genes superoxide dismutase 2 (Sod2)^25^. Similarly, FGF21 induced these genes, again suggesting the shared regulatory mechanisms between βOHB and FGF21.

We next examined the effects of βOHB and FGF21 on the molecular signatures of cardiac hypertrophy (Fig. 3B). βOHB stimulation reduced the expression of atrial natriuretic peptide (ANP), B-type natriuretic peptide (BNP), transforming grow factor-β (TGF-β) and connective tissue growth factor (CTGF), suggesting the protective role of βOHB and FGF21 against cardiac hypertrophy.

### AMPKα1 mediates βOHB- and FGF21-induced signaling pathways

To determine the upstream mechanisms by which βOHB and FGF21 induce FGF21 and PPARα mRNA expression, we performed a series of western blots. We found that not only βOHB and FGF21 but also AICAR (AMPK activator), norepinephrine (NE), and pemafibrate (selective PPARα modulator) increased FGF21 and PPARα protein levels. All of the compounds tested also increased the phosphorylation of LKB1 (an upstream kinase for AMPKα at Thr^172^)^21^, PGC1α and Sirt1 protein levels.

Then, the role of AMPKα was tested using siRNA-mediated AMPKα1 knockdown. AMPKα1-knockdown blunted an induction of FGF21, p-LKB1, PPARα, PGC1α and Sirt1 by each compound, while p-mTOR protein levels were increased (Fig. 3C). These results suggest that AMPK1α1 plays a critical role in the signaling pathways evoked by βOHB and FGF21. Full-length representative western blots are shown in Supplementary Fig. S2.

### β-klotho mediates FGF21-induced signaling pathway

To ascertain the role of β-klotho, we tested the effects of β-klotho knockdown on FGF21-induced gene expression in isolated cardiac myocytes. qPCR showed that transduction of cardiac myocytes with siRNA for β-klotho resulted in a three-fold reduction of β-klotho mRNA levels and more importantly, it completely abolished the FGF21 induction of PPARα, PGC1α, catalase, and Sod2 expression (Fig. 3D). Although β-klotho protein expression was not detected by Western blots in either cultured neonatal rat cardiac myocytes or mice heart tissues, these qPCR results suggest that FGF21 evokes intracellular signaling through β-klotho in cardiac myocytes.

### Enhanced elevation of serum βOHB and PPARα expression in Fgf21^−/−^ mice hearts during fasting

To determine the in vivo relevance of the role of FGF21 in the regulation of metabolic gene expression in the heart, we generated global FGF21 knockout mice (Fgf21^−/−^) by using a clustered interspaced short palindromic repeats (CRISPR)/Cas genome-editing technology^28^. Two single guide RNAs (sgRNAs) targeting upstream of exon 1 and downstream of exon 3 (Fgf21 exonic region) were designed and injected alongside Cas9 protein into pronuclear stage embryos zygotes. Mice heterozygous for the Fgf21^+/−^ allele were then intercrossed to generate FGF21^−/−^ mice. FGF21^−/−^ mice were born at the expected Mendelian ratio and were viable. Under basal conditions, Fgf21^−/−^ mice did not show any significant change in body weight, heart weight/body weight ratio (HW/BW) (Supplementary Table 2), although previous study showed an increase in heart weight/body weight ratio (HW/BW) in Fgf21^−/−^ mice^11^.

**Figure 4:**
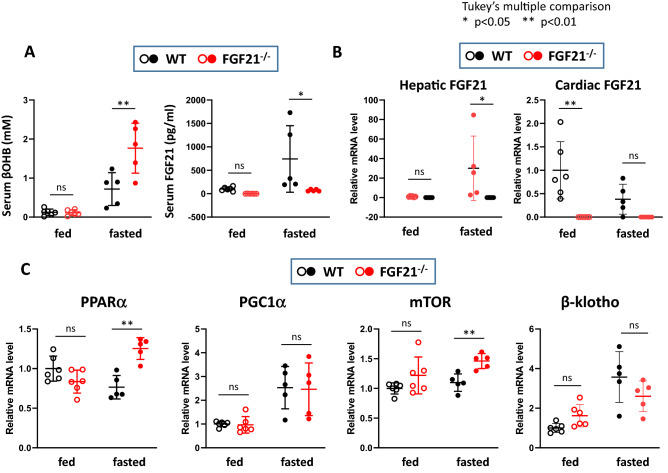
Effects of fasting on serum βOHB and nutrient signal-related gene expression in WT and Fgf21^−/−^ mice hearts. (**A**) ELISA was performed to measure the serum levels of βOHB and FGF21 in WT and Fgf21^−/−^ mice under either fed or 24 h-fasted conditions. (**B**) qPCR was performed to measure hepatic and cardiac FGF21 mRNAs in WT and Fgf21^−/−^ mice under either fed or 24 h-fasted conditions. (**C**) qPCR was performed to measure PPRAα, PGC1α, mTOR, and β-klotho mRNAs in WT and Fgf21^−/−^ mice under either fed or 24 h-fasted conditions. Data are mean ± SD (n = 5–6). *p < 0.05 and **p < 0.01 vs corresponding control (WT) analyzed by one-way ANOVA followed by Tukey correction for multiple comparisons.

Then, we subjected these mice to a 24 h-fasting challenge and explored changes in the expression of various genes in the heart. As expected, serum βOHB and FGF21 concentrations were robustly elevated in response to fasting in wild-type (WT) mice (Supplementary Table 2, Fig. 4A). Notably, serum βOHB levels were significantly higher in Fgf21^−/−^ mice than WT mice (Fig. 4A). Interestingly, while hepatic FGF21 mRNA levels were significantly increased after fasting as shown in previous studies^17,29^, cardiac FGF21 mRNA levels were not changed or rather decreased (Fig. 4B). This finding was in sharp contrast to the initial view that FGF21 is a ketogenic factor in response to fasting^15,17,29^. Hotta et al., on the other hand, have reported an increase in serum βOHB in Fgf21^−/−^ mice after fasting^30^.

PPARα expression was significantly higher in Fgf21^−/−^ mice than WT mice after fasting (Fig. 4C). mRNA levels for PGC1α, powerful regulators of mitochondrial function and biogenesis^31^, were similarly induced by fasting in Fgf21^−/−^ and WT mice. mTOR expression was significantly increased in Fgf21^−/−^ mice. mRNA levels of β-klotho, a co-receptor required for FGF21-mediated tissue-response^32^, were increased by fasting. This finding conforms to the previous studies demonstrating that the heart is a target of systemic FGF21. We need to await further work to ensure that β-klotho plays a crucial role in FGF21 signaling under the fasting condition.

### Enhanced expression of the oxidative response genes in Fgf21^−/−^ mice heart

**Figure 5:**
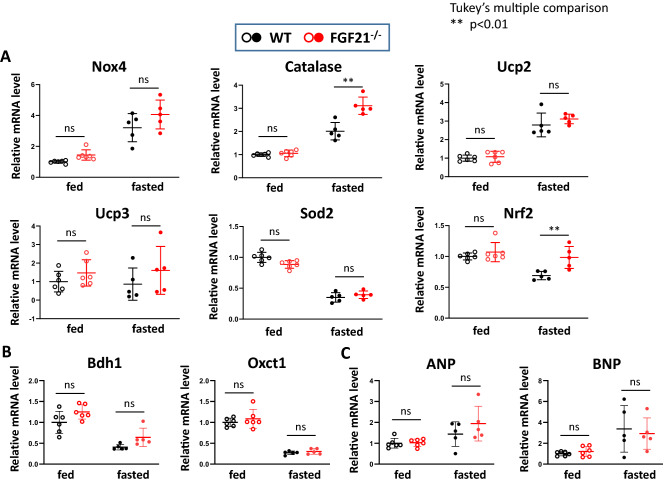
Effects of fasting on the oxidative stress response gene expression in Fgf21^−/−^ mice hearts. qPCR was performed to measure the mRNAs levels for oxidative stress response genes (**A**) and ketolytic enzymes and hypertrophic marker genes (**B**) in WT and Fgf21^−/−^ mice hearts under either fed or 24 h-fasted conditions. Data are mean ± SD (n = 5–6). **p < 0.01 vs corresponding control (WT) analyzed by one-way ANOVA followed by Tukey correction for multiple comparisons.

In both WT and Fgf21^−/−^ mice, the expression of the multiple genes involved in antioxidant system was significantly induced by fasting in the heart (Fig. 5A). They include Nox4, a major isoform of the NADPH oxidase family that is localized in mitochondria^33^, catalase, and uncoupling protein-2 (Ucp2), which attenuates mitochondrial reactive oxygen species (ROS) production by modulating proton leakage^34^. In contrast, expression of mitochondrial manganese-containing superoxide dismutase-2 (Sod2, or Mn-SOD) was clearly reduced after a 24-h fast. Interestingly, expression of catalase and Nrf2, a master regulator for antioxidant genes^35^, was significantly higher in Fgf21^−/−^ mice than WT mice (p < 0.01). In addition, Nox4, Ucp2 and Ucp3 tended to be more induced after fasting in Fgf21^−/−^ mice than WT mice although a difference did not reach statistical significance. These results suggest that oxidative stress response occurs in the heart after a 24-h fast, and this response is more prominent in Fgf21^−/−^ mice than in WT mice.

Contrary to our expectations, expression of the Bdh1 and the Oxct1 genes was downregulated by fasting in both WT and Fgf21^−/−^ hearts (Fig. 5B) despite an increase of serum βOHB levels (Fig. 4A), suggesting that the increased serum βOHB by itself does not induce ketone oxidation in the heart. qPCR analysis also revealed that an induction of the expression of hypertrophic marker genes^36^ such as ANP and BNP by fasting was similar between the two genotypes (Fig. 5C). The expression levels of other hypertrophic markers such as CTGF and angiotensin converting enzyme (ACE) were also similar in both mice (data not shown). These findings suggest that hypertrophic response is not affected by FGF21 signaling in the nutrient-stressed heart.

### Quantitative relationships between βOHB and oxidative stress response gene expression

**Figure 6:**
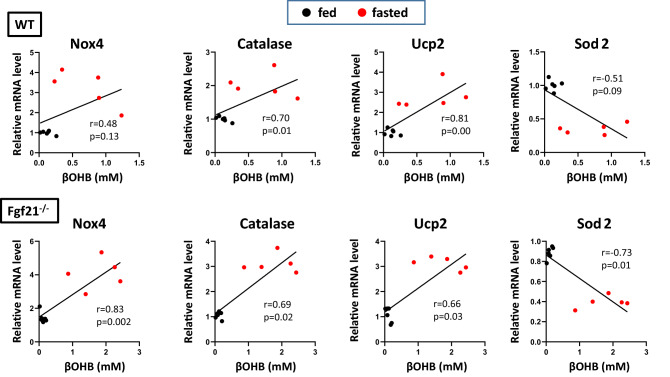
Correlations of the oxidative stress response gene expression with βOHB in Fgf21^−/−^ mice hearts. Linear gene expression values for Nox4, catalase, Ucp2 and Sod2 are shown in the *y*-axis in relative fluorescence units, whereas βOHB concentrations are shown in the *x*-axis. Pearson’s correlation coefficient and p-value are shown.

**Figure 7:**
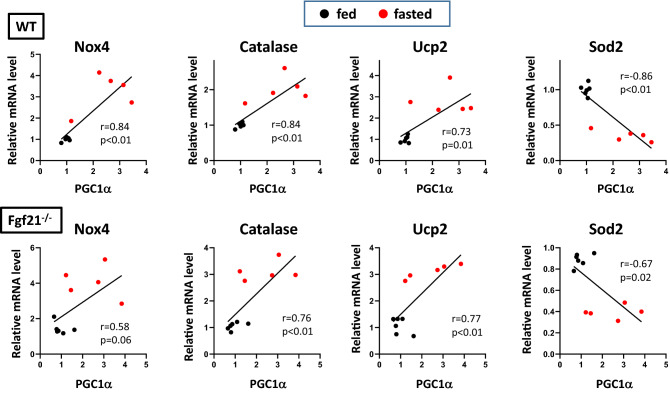
Correlations of the oxidative stress response gene expression with PGC1α in Fgf21^−/−^ mice hearts. Linear gene expression values for Nox4, catalase, Ucp2 and Sod2 are shown in the *y*-axis in relative fluorescence units, whereas PGC1α expression levels are shown in the *x*-axis. Pearson’s correlation coefficient and p-value are shown.

To assess the role of βOHB as a signal to induce fasting response in the heart, we examined the quantitative relationships between serum βOHB concentrations and oxidative stress response gene expression. Nox4 and antioxidant genes such as catalase and Ucp2 showed a highly significant correlation with βOHB concentrations in both WT and Fgf21^−/−^ mice hearts (Fig. 6). Similarly, the expression of these genes was significantly correlated with PGC1α expression levels in the hearts in both genotypes (Fig. 7). These results suggest that enhanced mRNA levels for oxidative response genes in Fgf21^−/−^ mice after a 24-h fast were at least partly attributed to increased βOHB levels and PGC1α levels. Interestingly, the Sod2 gene expression was robustly repressed by fasting, and its mRNA levels were inversely correlated with either serum βOHB or PGC1α expression levels (Figs. 6, 7).

### Expression of oxidative stress response genes in cardiac myocyte-specific knockout of Fgf21

**Figure 8:**
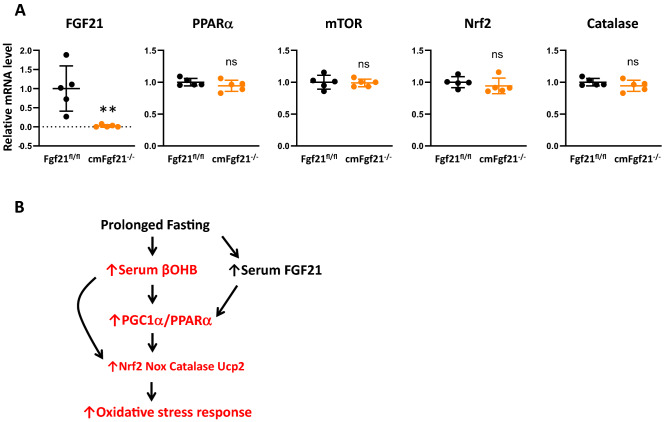
Cardiac gene expression in response to fasting in cmFgf21^−/−^ mice. (**A**) qPCR analysis of FGF21, PPARα, mTOR, Nrf2, and catalase mRNA levels in the heart in cmFGF21^−/−^ and FGF21^fl/fl^ mice subjected to a 24-h fast. Data are mean ± SD (n = 5). **p < 0.01 vs control analyzed by unpaired Student’s t-test. (**B**) Proposed scheme for the role of βOHB and FGF21 in the regulation of oxidative stress response gene expression during fasting in the heart. Prolonged fasting increases circulating levels of βOHB and FGF21, which promote Nrf2 and its downstream oxidative response genes (Nox4, catalase, Ucp2), partly through the PGC1α/PPARα signaling pathway. In Fgf21^−/−^ mice, such a response is induced to a greater extent by more increased levels of serum βOHB. Pathway promoted in Fgf21^−/−^ mice is indicated by red.

Although mainly being a hepatic hormone, FGF21 is also produced in the heart^11,37^. It has been proposed to exert its effects on the heart in a paracrine/autocrine fashion under pathophysiological conditions^37^. However, direct evidence to support this hypothesis has yet to be reported. To determine the impact of a loss of heart-derived FGF21 on the alteration of gene expression in the heart, we generated cardiomyocyte-specific Fgf21 knockout (cm*Fgf21*^−/−^) mice by crossing mice homozygous for the Fgf21-loxP–targeted allele (Fgf21^fl/fl^) with transgenic mice expressing Cre from the α-MHC promoter^38^. As expected, cmFgf21^−/−^ mice heart showed a significant suppression of Fgf21 compared with WT (Fgf21^fl/fl^) (Fig. 8A). Serum levels of βOHB, triglycerides, NEFAs as well as FGF21 were comparable between cmFgf21 and Fgf21^fl/fl^ in both fed and fasted states (Supplementary Table 2). Additionally, expression of the cardiac genes involved in fasting response and energy metabolism including PPARα, mTOR, Nrf2, and catalase was comparable between the two genotypes (Fig. 8A). These data suggest that autocrine/paracrine function of heart-derived FGF21 is dispensable for fasting-induced oxidative stress response in the heart.

## Discussion

In this work, we demonstrate four important points. First, both βOHB and FGF21 activate LKB1/AMPK/PGC1α/PPARα/Sirt1 signaling as well as PPARα activity, and upregulate the expression of the genes for oxidative stress response genes and downregulate the genes for hypertrophy markers in cultured cardiac myocytes. Second, unexpectedly, Fgf21^−/−^ mice have significantly higher serum βOHB concentrations than WT mice. Third, Fgf21^−/−^ mice hearts displayed higher expression of PPARα, Nrf2 and catalase genes after fasting than WT mice. Lastly, serum βOHB as well as PGC1α expression levels are positively correlated with the expression of oxidative stress response genes in the heart of both genotypes. From these data, we postulate that βOHB induces the oxidative stress response through the activation of PGC1α/PPARα signaling pathway, and circulating FGF21 plays a role as a regulator of such a fasting response by preventing excessive ketosis (Fig. 8B).

In the mouse model, expression of Bdh1 and Oxct1, the key enzymes for ketone oxidation in the heart, was significantly downregulated in the heart under fasting conditions where serum βOHB levels were increased (Fig. 5B). Thus, it is unlikely that βOHB alters the cardiac gene expression through the mechanism dependent upon the ketone oxidation and downstream metabolites including acetyl-CoA, succinyl-CoA, and NAD+ (nicotinamide adenine dinucleotide). Instead, it is conceivable that βOHB alters the myocardial gene expression through the epigenetic or posttranslational modification of the transcription factors, because βOHB acts as an endogenous inhibitor of class I HDACs as reported by Shimazu et al.^6^. They also reported that βOHB induced the expression of the oxidative stress resistance genes in HEK293 cells and mice kidney tissue. Consistently, we found that βOHB induced a subset of genes relevant to oxidative stress in the cardiac myocytes. Further, similar to βOHB in the present study, a previous study showed that HDAC inhibitors exert their anti-hypertrophic effects in cultured cardiac myocytes^39^. These results conform to the notion that βOHB alters gene expression through HDAC inhibition. To date, however, little is known how βOHB and HDAC inhibitors selectively alter only a subset of genes. Future studies investigating the role of βOHB as an HDAC inhibitor in the activation of LKB1-AMPK-PGC1α-PPARα pathway should provide a novel insight into the mechanisms that link βOHB to control of gene expression.

We demonstrate that βOHB phosphorylates LKB1 and AMPKα at Thr^172^. AMPK plays a key role as a cellular energy sensor, and LKB1 directly phosphorylates AMPKα at Thr^172^, and activates AMPK activity^21^. The classical view is that AMPK complexes are primarily activated when the availability of ATP is reduced^40^. However, it seems unlikely that βOHB depletes cellular ATP in cardiac myocytes because βOHB is used as an oxidative substrate to support ATP production^41^. Indeed, a recent study using perfused hearts showed that cardiac ketone oxidation rates are enhanced by increasing levels of βOHB in perfusate without declining either glucose or fatty acid oxidation rates^42^. We assume that βOHB promotes non-canonical AMPK activation. Previous seminal study demonstrated that mitochondrial ROS produced as the byproducts of aerobic metabolism acts as a physiological trigger for AMPK activation^43^. In line with this notion, mitochondrial ROS generated during respiration are likely to trigger AMPK activation in cardiac myocytes.

Previous studies showed that the heart synthesizes and releases FGF21, and FGF21 knockout mice exhibit increased cardiac hypertrophy and proinflammatory cytokine production in response to isoproterenol infusion, suggesting the cardioprotective role of FGF21^11,37^. However, the precise signaling pathways responsible for potential cardioprotective function of FGF21 remain unclear. We herein show that FGF21 activates AMPK-PPARα signaling but not just acts downstream of PPARα in cardiac myocytes.

Perhaps the most surprising finding is that PPARα expression is paradoxically increased in Fgf21^−/−^ mice despite the fact that FGF21 significantly activates PPARα expression and activity in vitro. How do we reconcile these findings? Fasting-induced βOHB levels in Fgf21^−/−^ mice were significantly higher than those in WT mice (Supplementary Table 2). In addition, Pearson’s correlation analysis showed that PPARα expression levels were significantly correlated with serum βOHB in Fgf21^−/−^ mice (r = 0.74, p = 0.01). These data suggest that an increase in PPARα expression in FGF21^−/−^ mice hearts is ascribed at least partly to an increased βOHB. However, taking the fact that serum βOHB levels were highly variable among fasted mice, further studies are required to test this hypothesis.

Prolonged fasting is a bioenergetic challenge for the organ systems including the heart which undergoes the activation of signaling pathways that support mitochondrial biogenesis to restore the nutrient balance^44^. While most of ROS in the cardiac myocytes is produced nonenzymatically by leakage of electron from mitochondrial electron transport chain during oxidative phosphorylation^45^, ROS is actively produced through ROS-generating systems under fasting^25^. Because mitochondrial Nox4 has been reported to represent a major source of oxidative stress under such a condition^33^, it is reasonable to speculate that upregulation of Nox4 expression in Fgf21^−/−^ mice hearts may represent the increase in mitochondrial oxidative stress in these mice, and thus upregulation of catalase and Ucp2 expression is likely to be caused by ROS generated by Nox4. Interestingly, Sod2 gene expression was significantly suppressed by fasting in our study. Nox4 generates charactaristically hydrogen peroxide as a major product while other Nox enzymes primarily generate superoxide (O_2_^−^)^46^. It is intriguing to speculate that hydrogen peroxide generated by Nox4 represses Sod2 gene expression by feedback mechanism. Further study is needed to test this assumption.

We showed that myocardial expression of Nox4, catalase, and Ucp2 was significantly correlated with βOHB and PGC1α. In addition, AICAR (AMPK activator) (Supplementary Fig. S3) or permafibrate (selective PPARα modulator) (Supplementary Fig. S4) significantly increased the expression of these genes in cardiac myocytes*.* These results suggest that Nox4, catalase and Ucp2 genes are direct or indirect targets of AMPK/PGC1α/PPARα signaling and an increased βOHB drives the higher expression of these oxidative stress response genes in Fgf21^−/−^ mice. Indeed, previous study showed that catalase gene promoter contains functional PPRE^47^. The catalase gene expression may represent a compensatory mechanism against the overproduction of H_2_O_2_ in Fgf21^−/−^ mice hearts. It should be mentioned that mTOR expression was significantly higher in Fgf21^−/−^ mice, and was significantly correlated with oxidative stress response gene expression (Supplementary Fig. S4). Given that Nox4 induces mTOR signaling leading to cardiac hypertrophy and fibrosis^48^, an observed increase in mTOR mRNA levels in Fgf21^−/−^ mice is likely to be the consequence of Nox4 activation.

Our impetus for this study is based on the hypothesis that βOHB and FGF21 act as signaling molecules regulating the cardiac metabolism under nutrient stress conditions including heart failure. Given that PPARα plays such a central role in energy metabolism of the heart^18^, we ask if βOHB- and FGF21-evoked signals regulate PPARα activity in the heart. Our findings that βOHB and FGF21 activate AMPK/PGC1α/PPARα signaling, and loss of FGF21 exaggerates serum βOHB elevation and promotes oxidative stress response in the heart contribute to the understanding of pathophysiological aspects of elevated serum βOHB and FGF21 in heart failure. Our findings that βOHB and circulating FGF21 control oxidative stress response induced by nutrient stress open new possibilities of βOHB and FGF21 for potential pharmacological intervention for heart failure in which elevated ROS production is believed to play a key role.

## Conclusions

This study demonstrates that βOHB and FGF21 activate AMPK/PGC1α/PPARα signaling pathways in cardiomyocytes. Global but not cardiomyocyte-specific knockout of FGF21 enhances the expression of antioxidant genes after a prolonged fast in mice. Our data underscore the importance of βOHB and circulating FGF21 as regulators of oxidative stress response in the heart by controlling PGC1α/PPARα signaling pathway. Given that oxidative stress is a key driver of heart failure and clear relevance to human pathologies^49^, our findings suggest that increasing delivery of βOHB and FGF21 to the heart seems to be a promising therapeutic approach to heart failure.

## Methods

An expanded methods section can be found in Supplementary Information.

### Animal care

The Institutional Animal Care and Use Committee of Gunma University Graduate School of Medicine approved all animal experiments (Approval number 17-039 for male mice and 19-047 for female mice). Animal experiments were performed to conform to the NIH guidelines describing Guide for the Care and Use of Laboratory Animals. All of the authors complied with the ARRIVE guidelines. Female mice of 3–4 weeks old were subjected to oocytes isolation for in vitro fertilization with spermatozoa from C57BL/6J male mice, and were euthanized via cervical dislocation. All other experiments were performed using male mice aged 7–12 weeks to avoid any confounding effects related to the estrogen cycle. All male mice strains used in this study were backcrossed to C57Bl/6J for at least three generations. The mice were housed in a temperature-controlled room (20–26 °C) with a 12-h light/12-h dark cycle and given unrestricted access to water and standard chow (CE-2, Clea Japan, Inc.). Euthanization of male mice was performed under 2% isoflurane anesthesia by intracardiac injection of 200 mL 5% potassium chloride to induce cardiac arrest.

### Preparation of neonatal rat cardiac myocytes

Cardiac myocytes were isolated from the hearts of 1- to 3-day-old neonatal rat pups and plated as previously described^50^. Using this method, we routinely obtained cardiac myocyte-rich cultures with > 95% of the cells being cardiac myocytes, as assessed by immunocytochemical staining with a monoclonal antibody against sarcomeric α-actinin (Sigma-Aldrich). Serum-starved cardiac myocytes with 0.1% BSA were incubated for 24 h in the indicated concentrations of βOHB (0–5 mM) and recombinant FGF21 (10 ng/mL, 100 ng/mL).

### Plasmids, transient transfection and luciferase assay

PPAR-responsive element (PPRE) × 3-TK-Luc, pCMX-Gal4-PPARα-LBD, pCMX-Gal4-PPARδ-LBD, pCMX-Gal4-PPARγ-LBD, and (UASG) × 4-TK-Luc reporter plasmid have been described^23^. Transient transfection of these plasmids were performed as described^51^. After 24 h, cells were stimulated with βOHB, recombinant FGF21, or pemafibrate for 24 h and harvested with Passive Lysis buffer (Promega). Luciferase activity was measured with luciferase assay substrate (Promega) using luciferase reporter assay system (Promega) as described previously^51^.

### Quantitative RT-PCR (qPCR)

Total RNA was isolated from left ventricles or cultured cardiac myocytes and cDNA was synthesized using Oligo(dTs) and M-MLV reverse transcriptase (Promega, UK). Real time (RT)-PCR was performed with the StepOnePlusTM System (Applied Biosystems, UK) using SYBR Green. Delta delta Ct values were calculated using Nuclear single-copy housekeeping gene 36B4 as a reference gene. Primer sequences are shown in Supplementary Table 1.

### Western blot analysis

Western blot analysis was performed according to standard procedures using the following primary antibodies: rabbit monoclonal FGF21 (Abcam, ab171941, 1:250), phospho-AMPKα (Thr^172^; Cell Signaling Technology, #2535, 1:250), AMPK (Cell Signaling Technology, #2603, 1:500), phospho-AMPKα1/α2 (Ser^485^/Ser^491^; Cell Signaling Technology, #4185, 1:250), phospho-mTOR (Ser^2448^; Cell Signaling Technology, #5536, 1:250), mTOR (Cell Signaling Technology, #2983, 1:500), phospho-LKB1 (Ser^428^; Cell Signaling Technology, #3482, 1:250), LKB1 (Cell Signaling Technology, #3047, 1:500), PPARα (Abcam, ab24509, 1:250), PGC1α (Cell Signaling Technology, #2178, 1:250), ACC (Cell Signaling Technology, #3676, 1:500), rabbit polyclonal phospho-ACC (Ser^79^; Cell Signaling Technology, #3661, 1:250), SIRT1 (Cell Signaling Technology, #9475, 1:250), β-actin (Cell Signaling Technology, #4970, 1:500), and GAPDH (Cell Signaling Technology, #2118, 1:500). Antigens were revealed by Immobilon Western HRP Substrate (Millipore) after an incubation with horseradish peroxidase-conjugated anti-rabbit or mouse IgG. The blots were exposed on the autoradiography film then developed with Fuji Medical Film Processor FPM100, changed to the appropriate grey background using Microsoft PowerPoint. The density of a band was quantified using ImageJ software.

### siRNA-mediated interference

Cardiomyocytes were transfected with siRNA designed to silence GFP (siGFP) (Santa Cruz Biotechnology, sc-45924, 50 nM) or siAMPKα1 (sc-270142, 50 nM) and siGFP (Stealth RNAi Negative Control Duplexes, Medium GC Duplex, 20 nM) or siKLB (Silencer Select Pre-designed siRNA: s144500 and s144502, 5 nM each) with Lipofectamine RNAiMAX Reagent (Invitrogen) according to the manufacturer’s protocol. After a 24 h transfection, the cells were exposed to various treatments and then collected for analysis of luciferase assays and extracted RNA.

### CRISPR-Cas9-mediated deletion of the Fgf21 gene in vivo

The global Fgf21^−/−^ mice were generated by using a CRISPR/Cas9 genome-editing technology onto pronuclear stage embryos^28^. In brief, female C57BL/6J mice (Japan SLC Inc., Tokyo, Japan) were super-ovulated by intraperitoneal injection of 7.5 units of pregnant mare’s serum gonadotropin (PMSG; ASKA Pharmaceutical Co., Ltd., Tokyo, Japan), followed by 7.5 units of human chorionic gonadotropin (hCG; ASKA Pharmaceutical) 48 h later, as previously described^52^. Additional details are described in Supplementary material.

### Cardiac myocyte-specific deletion of Fgf21 (cmFgf21^−/−^)

To generate mice with a cardiomyocyte-specific deletion of Fgf21 (cmFgf21^−/−^), we crossed mice carrying Fgf21-flox alleles with transgenic mice harboring Cre recombinase driven by a α-myosin heavy chain (α-MHC) promoter described previously^38^. All mice were fed normal chow diets and housed under standard light–dark cycled conditions.

### Statistical analysis

All continuous variables are presented as the mean ± standard deviation (SD), unless otherwise specified. A two-group comparison was performed using the unpaired Student’s t-test, while a multiple-group comparison was performed by one-way ANOVA with Dunnett or Tukey correction. Two-sided p-value < 0.05 was considered statistically significant. *p < 0.05, **p < 0.01.

## Supplementary Information

Supplementary Information.

## Data Availability

The datasets used and/or analysed during the current study available from the corresponding author on reasonable request.
