# Supplementary material for: Ketone body and FGF21 coordinately regulate fasting-induced oxidative stress response in the heart
Source: Sci Rep. 2022 May 5;12:7338. doi: 10.1038/s41598-022-10993-4 (PMC9072431; doi:10.1038/s41598-022-10993-4)
Supplement: Supplementary file 1 — Supplementary Information. [file 41598_2022_10993_MOESM1_ESM.docx]

**Supplementary Material**

**Ketone body and FGF21 coordinately regulate fasting-induced**

**oxidative stress response in the heart**

Ryo Kawakami^1†^, Hiroaki Sunaga^1,2†^, Tatsuya Iso^1^, Ryosuke Kaneko^3,4^, Norimichi Koitabashi^1^,

Masaru Obokata^1^, Tomonari Harada^1^, Hiroki Matsui^5^, Tomoyuki Yokoyama^5^, and Masahiko Kurabayashi^1^*

^1^Department of Cardiovascular Medicine, Gunma University Graduate School of Medicine, Maebashi, Gunma, Japan; ^2^Center for Liberal Arts and Sciences, Ashikaga University, 268-1 Omae-machi, Ashikaga, Tochigi, 326-8558, Japan; ^3^Bioresource Center, Gunma University, Graduate School of Medicine, Maebashi, Gunma, Japan; ^4^ Osaka University, Graduate School of Frontier Biosciences, 1-3 Yamadaoka, Suita, Osaka, Japan; ^5^Department of Laboratory Sciences, Gunma University Graduate School of Health Sciences, Maebashi, Gunma, Japan;

†　The first two authors contributed equally to this work.

Short title: Ketone and FGF21 under cardiac nutrient stress

***Address for Correspondence**

Masahiko Kurabayashi, MD, PhD

Department of Cardiovascular Medicine, Gunma University Graduate School of Medicine,

3-39-15 Showa-machi, Maebashi, Gunma 371-8511, Japan

Tel.: +81-27-220-8140; Fax: +81-27-220-8150; E-mail address: mkuraba@gunma-u.ac.jp

**1. Methods**

**CRISPR-Cas9-mediated deletion of the Fgf21 gene in vivo**

Introduction of Cas9 protein, guide RNA, and single strand oligodeoxynucleotide (ssODN) into pronuclear stage embryos was carried out using the TAKE method. {Kaneko, 2017 #401} Cas9 protein, guide RNA, and ssODN were purchased from IDT (Integrated DNA Technologies, Inc., Coralville, IA, USA). Mixture of crRNA and tracrRNA was used as guide RNA. Guide RNAs were designed to delete exon 1, 2, and 3 of the Fgf21 gene of the C57BL/6 mouse (5’-CCCCATTGCATCATCCGTCC-3’ and 5’-CCTATATAATCCAACAATCG-3’). ssODN was designed to target the upstream and downstream sequences of Fgf21 gene of the C57BL/6 mouse (5’-CCCTTTTCATTCAGACCCCTGTTGGAAAGACCCCCCCATTGCATCATCCGTCGTGGTTACCCACTCTCTGTATCTTTGGCCTCCTGACTGCTGCACCTGG-3’). The CRISPR/Cas9 solution contained 100 ng/μL Cas9 protein, 66 ng/μL each crRNA, 133 ng/μL tracrRNA, and 150 ng/μL ssODN in Opti-MEM (Thermo Fisher Scientific Inc., MA, USA). Super electroporator NEPA21 (NEPA GENE Co. Ltd., Chiba, Japan) was used to introduce Cas9 protein, guide RNA, and ssODN into embryos. The poring pulse was set to voltage: 225 V, pulse length: 2.0 ms, pulse interval: 50 ms, number of pulses: 4, decay rate: 10%, polarity: +. The transfer pulse was set to a voltage: 20 V, pulse length: 50 ms, pulse interval: 50 ms, number of pulse: 5, decay rate: 40%, Polarity: +/-. The CRISPR/Cas9 solution (45 μL) was filled between metal plates of 5 mm gap electrodes on a glass slide (CUY505P5, NEPA GENE Co. Ltd.). The embryos placed in line between the electrodes were then discharged. The embryos were then cultured in HTF at 37 °C in 5% CO_2_/95% air. On the next day, two-cell embryos were transferred into the oviduct ampulla (18–24 embryos per oviduct) of pseudopregnant ICR (Japan SLC Inc.) females.

**2. Results**

**Supplementary Figure Legends**

**Suppl. Figure S1. Original western blot of Figure 1D.**

Representative western blots are shown. The blotted membranes were closely-cropped before hybridizing with the antibodies because hybridization conditions used did not give rise to signals in the range away from the expected molecular weights. Membrane edges were hardly visible in some blots because of the high signal to noise ratio. The blots were exposed on the autoradiography film then developed with Fuji Medical Film Processor FPM100, changed to the appropriate grey background using Microsoft PowerPoint. These images were inserted into Fig. 1D of the main article.

**
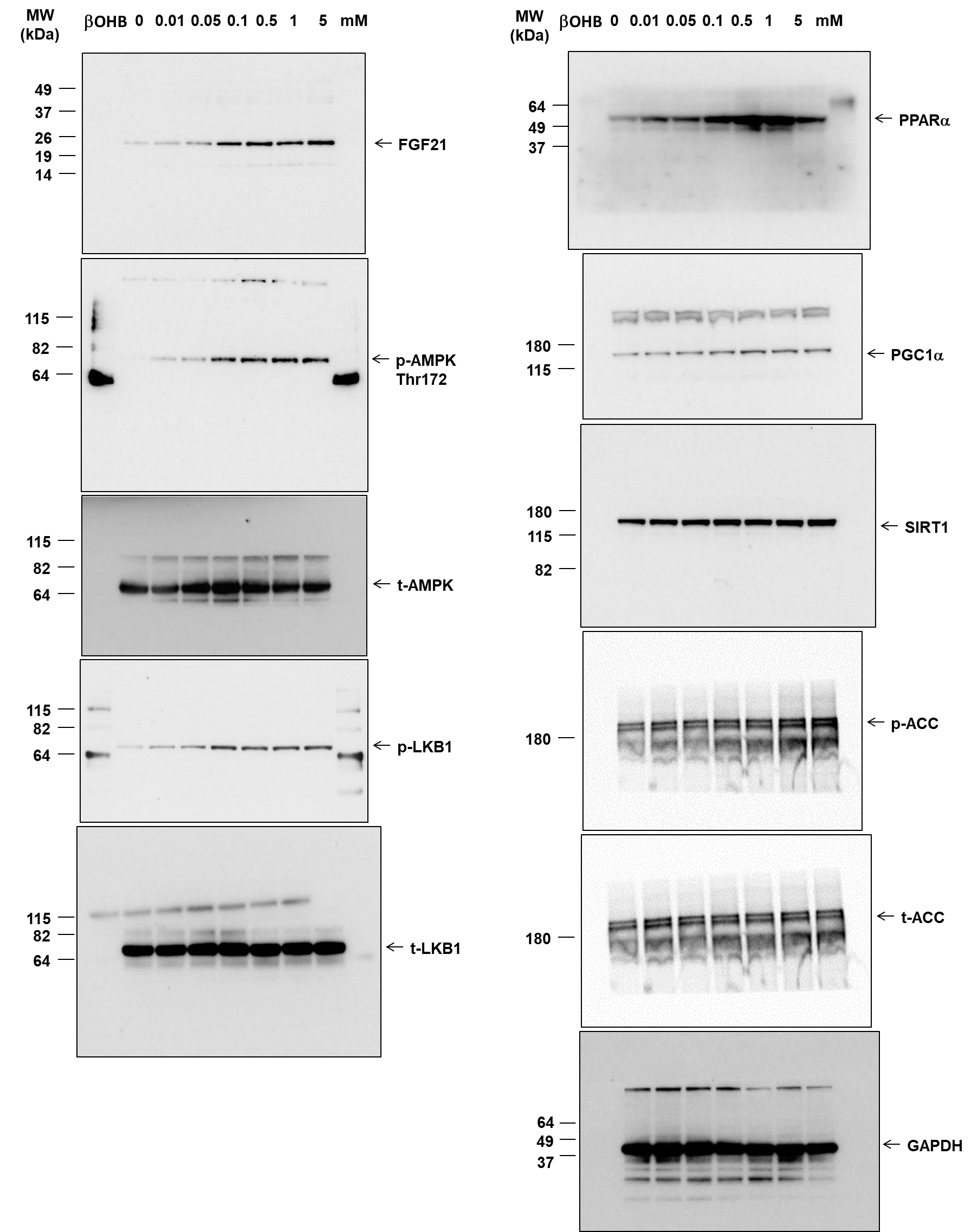
**

**Suppl. Figure S2. Original western blot of Figure 3C.**

Representative western blots are shown. The blotted membranes were closely-cropped before hybridizing with the antibodies because hybridization conditions used did not give rise to signals in the range away from the expected molecular weights. For the blots of t-AMPK and β-actin, membranes were hybridized with two antibodies simultaneously to be able to provide evidence that the same amounts of proteins were loaded in each lane. Membrane edges were hardly visible in some blots because of the high signal to noise ratio. Images of right several lanes were excluded from the original images because those were irrelevant to this study. The blots were exposed on the autoradiography film then developed with Fuji Medical Film Processor FPM100, changed to the appropriate grey background using Microsoft PowerPoint. These images were inserted into Fig. 3C of the main article.

**
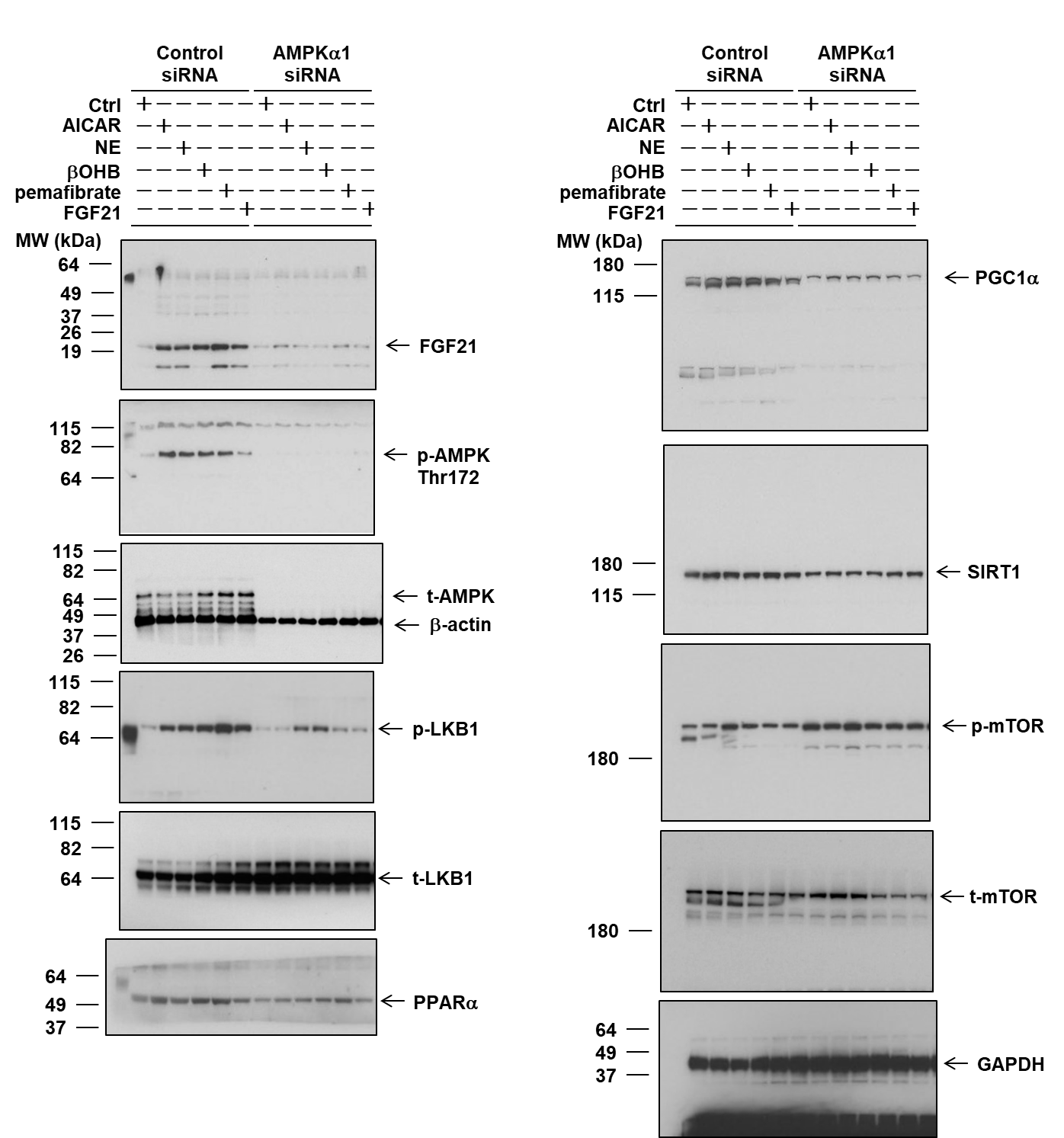
**

**Suppl. Figure S3. Effects of AMPK activator AICAR on gene expression in neonatal rat ventricular cardiac myocytes.** qPCR was performed to measure the mRNA levels of the indicated genes. Data are mean ±SD (n=6). **p<0.01 vs control analyzed by unpaired Student’s t-test.

**Suppl. Figure S4． Effects of selective PPARα modulator pemafibrate on gene expression in neonatal rat ventricular cardiac myocytes.** qPCR was performed to measure the mRNA levels of the indicated genes. Data are mean ±SD (n=6). **p<0.01 vs control analyzed by unpaired Student’s t-test.

**Suppl. Figure S5．Correlations of the oxidative stress response gene expression with mTOR in FGF21^-/-^ mice.** Linear gene expression values for Nox4, catalase, Ucp2 and Sod2 are shown in the *y*-axis in relative fluorescence units, whereas mTOR levels relative to WT are shown in the *x*-axis. Pearson’s correlation coefficient was shown.

**Suppl. Table 1**

**Primer sequences used for qPCR**

| **Primers set** | **Forward (5’-3’)** | **Reverse (5’ -3’)** |
| --- | --- | --- |
| **Fgf21 (rat)** | **GAGGCATACCCCATCTCTGA** | **GCAAAGGCTCTACCATGCTC** |
| **Pparα (rat)** | **GACAAGGCCTCAGGATACCA** | **GTCTTCTCAGCCATGCACAA** |
| **βkl (rat)** | **ATCTCCACTCTGGCTGCACT** | **CTCTGCTGTGCCCTTTCTTC** |
| **Nox4 (rat)** | **ACTGCCTCCATCAAGCCAAGA** | **GACTTCCAAATGGGCCATCAA** |
| **Nrf2 (rat)** | **GCTGCCATTAGTCAGTCGCTCTC** | **ACCGTGCCTTCAGTGTGCTTC** |
| **Sod2 (rat)** | **GACTAGGCCACAGGGCATTCA** | **ACTCAGAAACCCGTTTGCCTCTAC** |
| **Catalase (rat)** | **GAACATTGCCAACCACCTGAAAG** | **GTAGTCAGGGTGGACGTCAGTGAA** |
| **Anp (rat)** | **CCGAGACAGCAAACATCAGA** | **GGATCTTTTGCGATCTGCTC** |
| **Bnp (rat)** | **CAGAACAATCCACGATGCAG** | **CACTGTGGCAAGTTTGTGCT** |
| **Tgfβ (rat)** | **CATTGCTGTCCCGTGCAGA** | **AGGTAACGCCAGGAATTGTTGCTA** |
| **Ctgf (rat)** | **AAGACACATTTGGCCCTGAC** | **GTAATGGCAGGCACAGGTCT** |
| **36B4 (rat)** | **ATCCCTGACGCACCGCCGTGA** | **TGCATCTGCTTGGAGCCCACGTT** |
| **Fgf21 (mouse)** | **ACACTGAAGCCCACCTGGAGA** | **CTGCAGGCCTCAGGATCAAAG** |
| **Pparα (mouse)** | **CTCAGGGTACCACTACGGAGTTCAC** | **TGAATCTTGCAGCTCCGATCAC** |
| **Pgc-1α (mouse)** | **TTGACAGCTGCATTCATTTATCACC** | **AACACTTGAGCAAGCATTCGACA** |
| **Catalase (mouse)** | **TCTACACAAAGGTGTTGAACGAGGA** | **CCATAGTCAGGGTGGACGTCAG** |
| **Ucp2 (mouse)** | **ACTCTGCCTTGGGCCAGTA** | **GCTGCTCATAGGTGACAAACATC** |
| **Ucp3 (mouse)** | **CTCTGCACTGTATGCTGAAGATG** | **CACGTTCCAAGCTCCCAGA** |
| **Nox4 (mouse)** | **TATCTTGCACCAAACACAGAAGCAC** | **AAGCAAAGCAGGGTATCACTCCA** |
| **mTOR (mouse)** | **CCCGGACAAGGACAGACTCCTA** | **GGTTTCACCAAACCGTCTCCA** |
| **Nrf2 (mouse)** | **TCCGCTGCCATCAGTCAGTC** | **ATTGTGCCTTCAGCGTGCTTC** |
| **Sod2 (mouse)** | **GAGAATCTCAGTGCTCACTCGTGTC** | **GGAACCCTAAATGCTGCCAGTC** |
| **Anp (mouse)** | **CCTGTGTACAGTGCGGTGTC** | **AAGCTGTTGCAGCCTAGTCC** |
| **Bnp (mouse)** | **GCCAGTCTCCAGAGCAATTC** | **CCGATCCGGTCTATCTTGTG** |
| **Ctgf (mouse)** | **ACCCGAGTTACCAATGACAATACC** | **CCGCAGAACTTAGCCCTGTATG** |
| **Ace (mouse)** | **CTGATCACAGGCCAGCCTAACA** | **GTTCTCGGTGACGAGCCATTC** |
| **Gapdh (mouse)** | **TGTGTCCGTCGTGGATCTGA** | **TTGCTGTTGAAGTCGCAGGAG** |

**Suppl. Table 2**

**Body weight, heart weight and serum parameters in WT and Fgf21^-/-^ mice.** Data are mean ±SD (n=5-6). *p<0.05 and **p<0.01 vs corresponding control (WT) analyzed by one-way ANOVA followed by Tukey correction for multiple comparisons.

|  | **Fed** | | **Fasted** | |  |
| --- | --- | --- | --- | --- | --- |
|  | **WT**  (n=6) | **FGF21^-/-^**  (n=6) | **WT**  (n=5) | **Fgf21^-/-^**  (n=5) | **annotations** |
| **BW (g)** | 27.7 ± 1.4 | 25.7 ± 1.0 | 24.0 ± 3.4 | 23.2 ± 1.8 | none |
| **HW/BW (mg/g)** | 4.1 ± 0.1 | 4.2 ± 0.3 | 4.0 ± 0.5 | 4.1 ± 0.5 | none |
| **FGF21 (pg/mL)** | 101.5 ± 42.6 | 1.0 ± 1.8 | 742.0 ± 709.0 | 72.5 ± 21.3 * | * p=0.03 |
| **NEFA (mEq/L)** | 0.38 ± 0.06 | 0.35 ± 0.05 | 0.41 ± 0.46 | 0.37 ± 0.09 | none |
| **TG (mg/dL)** | 85.2 ± 27.1 | 71.1 ± 14.5 | 20.6 ± 11.7 | 28.3 ± 6.3 | none |
| **βOHB (mM)** | 0.12 ± 0.08 | 0.11 ± 0.07 | 0.72 ± 0.42 | 1.77 ± 0.64 ** | ** p=0.002 |
| **glucose (mg/dL)** | 174 ± 18 | 191 ± 33 | 71 ± 17 | 109 ± 25 | none |

**Suppl. Table 3**

**Body weight, heart weight and serum parameters in Fgf21^fl/fl^ and cmFgf21^-/-^ mice.** Data are mean ±SD (n=5). **p<0.01 vs control analyzed by unpaired Student’s t-test.

|  | **Fasted** | |  |
| --- | --- | --- | --- |
|  | **Fgf21^fl/fl^**  (n=5) | **cmFGF21^-/-^**  (n=5) | **p value** (unpaired t-test) |
| **BW (g)** | 22.8 ± 1.9 | 23.0 ± 1.4 | ns |
| **HW/BW (mg/g)** | 3.9 ± 0.1 | 4.0 ± 0.3 | ns |
| **FGF21 (pg/mL)** | 229.7 ± 118.5 | 313.1 ± 263.9 | ns |
| **NEFA (mEq/L)** | 0.62 ± 0.06 | 0.73 ± 0.17 | ns |
| **TG (mg/dL)** | 105.5 ± 21.1 | 104.8 ± 21.7 | ns |
| **βOHB (mM)** | 2.08 ± 0.53 | 2.33 ± 0.55 | ns |
| **glucose (mg/dL)** | 64.8 ± 11.6 | 65.0 ± 5.6 | ns |
